# Management of bleeding from a left-sided epiphrenic esophageal diverticulum: role of positional adjustment in clot removal

**DOI:** 10.1055/a-2587-9272

**Published:** 2025-05-09

**Authors:** Kohei Uyama, Yohei Yabuuchi, Yoshiki Morihisa, Soichiro Nagao, Shinsuke Akiyama, Shuko Morita, Tetsuro Inokuma

**Affiliations:** 1 26330 Department of Gastroenterology, Kobe City Medical Center General Hospital, Kobe, Japan

Epiphrenic esophageal diverticulum is a rare condition, typically located within 10 cm of the gastroesophageal junction and is commonly right-sided 1 2 . It is also an infrequent cause of upper gastrointestinal bleeding 3 . There is no established method for achieving hemostasis in esophageal diverticular bleeding 4 .

A 65-year-old man was admitted for treatment of a dissecting abdominal aortic aneurysm. During hospitalization, the patient developed hypotension, melena, and progressive anemia. Contrast-enhanced computed tomography (CT) revealed an esophageal diverticulum on the left side of the distal esophagus with active extravasation from this site ( Fig. 1 ). Esophagogastroduodenoscopy showed the diverticulum filled with large blood clots, obstructing visualization of its interior. Attempts to remove the clots with grasping forceps were unsuccessful ( Fig. 2 ). The patient’s position was then changed from a left lateral decubitus position to a right lateral decubitus position using gravity to facilitate clot removal. This maneuver cleared the blood clots, enabling full inspection of the diverticulum. An exposed vessel and nearby erosions were identified as the sources of bleeding ( Fig. 3 ). Endoscopic hemostasis was achieved using endoclips ( Fig. 4 , Video 1 ).

**Fig. 1:**
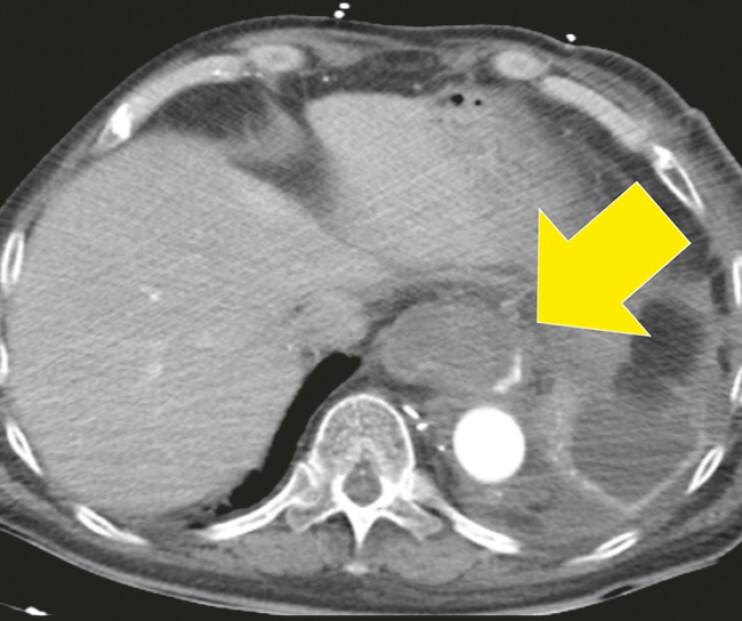
Contrast-enhanced computed tomography (CT) revealed an epiphrenic esophageal diverticulum on the left side of the distal esophagus, with active extravasation from the site (arrow).

**Fig. 2:**
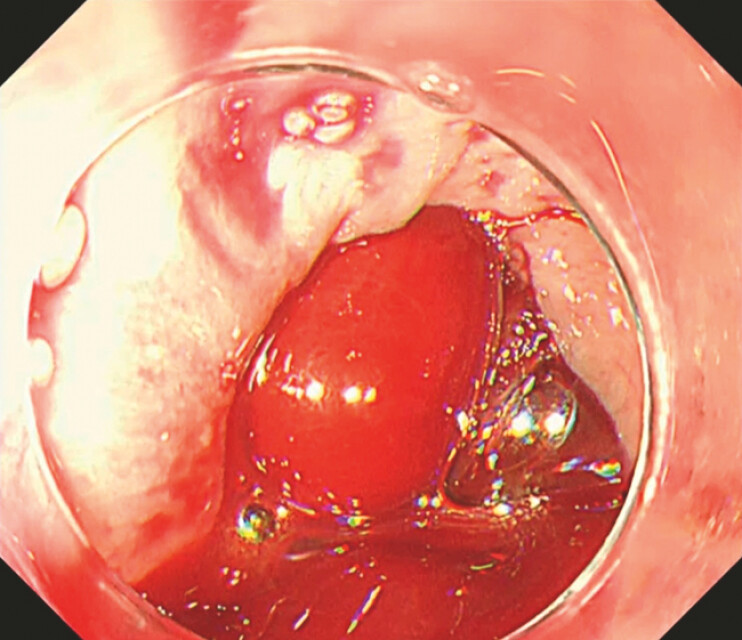
The esophageal diverticulum was filled with large blood clots, and removal of the clots using grasping forceps was difficult.

**Fig. 3:**
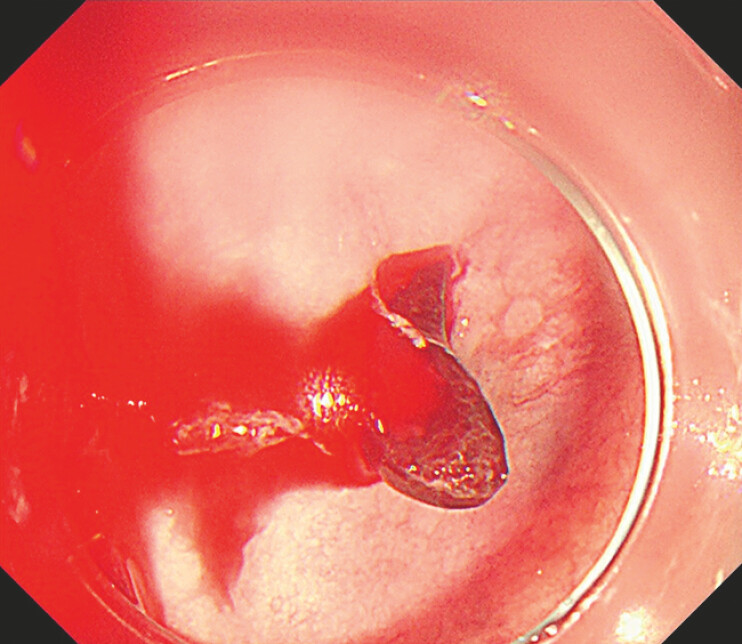
An exposed vessel was identified as the source of bleeding.

**Fig. 4:**
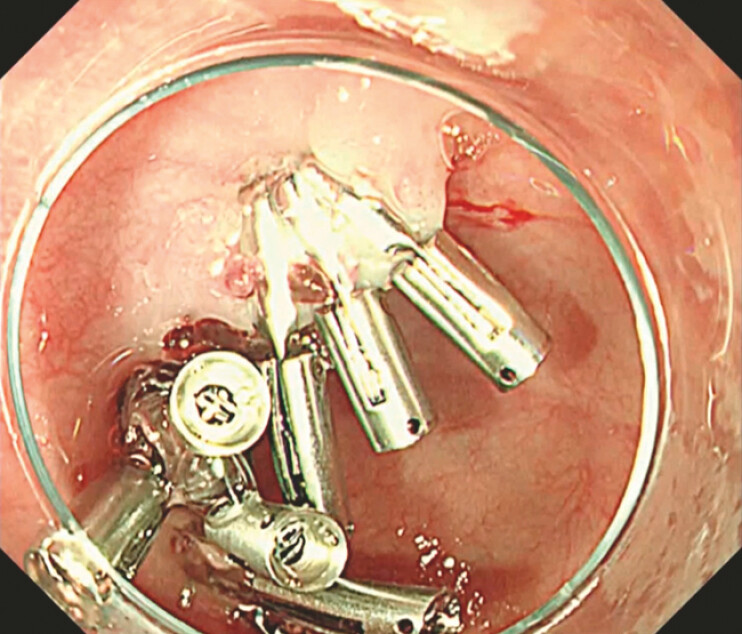
Hemostasis was achieved using clips.

Management of bleeding from a left-sided epiphrenic esophageal diverticulum: role of positional adjustment in clot removal.Video 1

This case highlights the rarity of bleeding from a left-sided epiphrenic esophageal diverticulum. In this case, the standard endoscopic position (left lateral decubitus) caused blood clots to pool in the diverticulum, obstructing the view. Changing to the right lateral decubitus position provided a simple and effective method to use gravity to clear the diverticulum without needing specialized tools. This positional adjustment proved crucial for visualizing the bleeding source and achieving hemostasis. Endoscopic treatment of bleeding from esophageal diverticula presents unique challenges owing to anatomical constraints and the risk of incomplete visualization. Positional changes are a valuable strategy for improving visibility in straight structures like the esophagus.

Endoscopy_UCTN_Code_TTT_1AO_2AD Endoscopy_UCTN_Code_CCL_1AB_2AD_3AZ

## Notes

Endoscopy E-Videos https://eref.thieme.de/e-videos
*E-Videos* is an open access online section of the journal *Endoscopy* , reporting on interesting cases and new techniques in gastroenterological endoscopy. All papers include a high-quality video and are published with a Creative Commons CC-BY license. Endoscopy E-Videos qualify for HINARI discounts and waivers and eligibility is automatically checked during the submission process. We grant 100% waivers to articles whose corresponding authors are based in Group A countries and 50% waivers to those who are based in Group B countries as classified by Research4Life (see: https://www.research4life.org/access/eligibility/ ). This section has its own submission website at https://mc.manuscriptcentral.com/e-videos .
